# Making sense of perceptions of risk of diseases and vaccinations: a qualitative study combining models of health beliefs, decision-making and risk perception

**DOI:** 10.1186/1471-2458-11-943

**Published:** 2011-12-20

**Authors:** Lyndal Bond, Terry Nolan

**Affiliations:** 1 MRC/CSO Social and Public Health Sciences Unit, Glasgow, UK; 2 School of Population Health, University of Melbourne, Melbourne, Australia

## Abstract

**Background:**

Maintaining high levels of childhood vaccinations is important for public health. Success requires better understanding of parents' perceptions of diseases and consequent decisions about vaccinations, however few studies have considered this from the theoretical perspectives of risk perception and decision-making under uncertainty. The aim of this study was to examine the utility of subjective risk perception and decision-making theories to provide a better understanding of the differences between immunisers' and non-immunisers' health beliefs and behaviours.

**Methods:**

In a qualitative study we conducted semi-structured in-depth interviews with 45 Australian parents exploring their experiences and perceptions of disease severity and susceptibility. Using scenarios about 'a new strain of flu' we explored how risk information was interpreted.

**Results:**

We found that concepts of dread, unfamiliarity, and uncontrollability from the subjective perception of risk and ambiguity, optimistic control and omission bias from explanatory theories of decision-making under uncertainty were useful in understanding why immunisers, incomplete immunisers and non-immunisers interpreted severity and susceptibility to diseases and vaccine risk differently. Immunisers dreaded unfamiliar diseases whilst non-immunisers dreaded unknown, long term side effects of vaccines. Participants believed that the risks of diseases and complications from diseases are not equally spread throughout the community, therefore, when listening to reports of epidemics, it is not the number of people who are affected but the familiarity or unfamiliarity of the disease and the characteristics of those who have had the disease that prompts them to take preventive action. Almost all believed they themselves would not be at serious risk of the 'new strain of flu' but were less willing to take risks with their children's health.

**Conclusion:**

This study has found that health messages about the risks of disease which are communicated as though there is equality of risk in the population may be unproductive as these messages are perceived as unbelievable or irrelevant. The findings from this study have implications beyond the issue of childhood vaccinations as we grapple with communicating risks of new epidemics, and indeed may usefully contribute to the current debate especially in the UK of how these theories of risk and decision-making can be used to 'nudge' other health behaviours.

## Background

Few would argue against the success of mass vaccination programmes in reducing and, in the case of smallpox, eliminating infectious diseases. Continued success however, requires adequate coverage which in turn requires parents to be committed to vaccination as an effective method of preventing their children from contracting diseases. Such commitment may be adversely affected by an increasingly complicated immunisation schedule for an increasing number of diseases and scares of vaccine safety (e.g. MMR debate that continues in the UK). It has also been argued that the very success of mass vaccination programmes has limited parents' experience of vaccine preventable diseases and thus affected their assessment of the severity of diseases and importance of prevention [1,2]. There is therefore, a continued need to better understand parents' perceptions of what is serious, what is risky and what is best for their children's health. Indeed with SARS, bird and swine flu, this extends to what people think about how best to protect their own health. Theories of health beliefs, decision-making and subjective risk perception have all been used in attempts to explain parents' decisions with respect to immunisation [3-5], but with limited success in explaining why parents differ in their perceptions of risk. With the exception of Hawe's et al research, [6] public health and health promotion campaigns have not drawn explicitly on or tested these theories. A recent review discussed how decision-making theories might help to explain parent attitudes and behaviour with respect to MMR vaccine uptake, but provided no primary evidence [7]. We argue in this paper that our understanding of immunisation choices and how we may best influence those choices may be increased through a synthesis of health beliefs, decision-making and subjective risk perception theories. This paper describes the findings from a qualitative study examining the utility of the risk perception and decision making theories to provide a better understanding of the differences between immunisers' and non-immunisers' health beliefs and behaviours, when considering the risks of a 'new strain of flu'.

### Health belief model

Theories of health protective behaviour offer an appealing framework in which to interpret differences in compliant and non-compliant parents with respect to childhood vaccinations [8]. While several models have been developed to account for people's adoption of health protective behaviour such as the Theory of Reasoned Action [9], the Triandis Model [10], Multi-Attribute Utility (MAU) Theory [11] and the Subjective Expected Utility Theory [8], the Health Belief Model is possibly the simplest and the most widely used and tested [12,13]. The four elements of the Health Belief Model are: perceived susceptibility (likelihood of getting the disease), perceived severity (perception of how serious an outcome or consequence is from the disease), perceived benefits (efficacy of preventive action undertaken) and perceived barriers (time, effort, money, inconvenience, pain, side effects of preventive action) [12,13].

Attempts to assess the association of these elements and childhood immunisation uptake have been inconclusive with some studies reporting expected relationships [14-16] and others contrary to what would be expected [17,18]. These contradictory findings have led to the conclusion that the health beliefs of mothers are not important contributors to immunisation uptake or completion and are less important than socio-demographic factors. That is, incomplete immunisation (fall behind the immunisation schedule or fail to complete) is associated with being poor [17], and being a single parent [19,20]. Low maternal education has usually been found to be a risk factor for not completing immunisation [20]. Being anti immunisation on the other hand, is associated with high education [21].

While in some instances theories of health protective behaviours have been shown to differentiate between complete, incomplete and non-immunisers, none of them provides an explanation of how people perceive risks or how their perceptions might influence behaviour. Indeed these models assume a rational basis for these decisions: a simple weighing up of information regarding severity, susceptibility, benefits and barriers. There is not, however, a simple relationship between mortality or morbidity figures and the perception of risk [22]. People do not perceive, interpret or act on risk information in the way expected by risk experts in general [22] nor specifically when considering vaccines and diseases [4,23].

### Subjective perceptions of risk and decision-making under uncertainty

Two domains which have addressed lay rather than expert perceptions of risk and what influences decisions encompassing risk are studies of the subjective perception of risk [24-26] and the study of decision-making under uncertainty (also referred to as the psychology of choice)[27,28]. Both approaches focus on risk as a subjective rather than an objective concept, and both involve social and psychological aspects that impact on the individual cognitive structure of risk perception.

Research into the *subjective perception of risk *generally involves asking study participants to rate a heterogeneous set of environmental or health risks including risks from individual activities, residential or work conditions, hazards from technologies, substances or products, and natural hazards. Respondents are asked to rate these activities or hazards in terms of perceived magnitude of risk, the acceptability of the risk and other aspects such as likelihood of death, catastrophic potential, avoidability, fear, familiarity, imposed or personal choice, time scale of impact, benefits of risk source, degree of concern, personal exposure etc.

Studies including vaccination (otherwise unspecified) as a hazard have reported vaccination as low risk [24,25]. Vaccination is generally considered to be a risk that is not 'dreaded' (controllable, not fatal, individual, low risk to future generations) but is somewhat 'unknown' (not observable, effect delayed, new risk, risk unknown to science). Slovic [25], in a study of risk perception of prescription drugs including vaccines, found vaccines were generally considered beneficial by the sample. However, people associating negative meanings to drugs tended to judge drugs and vaccines as having higher risks and lower benefits than people who associated positive meanings to drugs.

This research has consistently found that risks are perceived more negatively if exposure to the hazard is involuntary, people perceive they have little personal control over outcomes and there is uncertainty about the consequences of the outcome(s), the hazard is unfamiliar, the effects of the hazard are delayed, the hazard has catastrophic potential, the benefits are not immediately apparent and the hazard is caused by human rather than natural causes [24]. These have been summarised by two factors labelled 'Dread' (uncontrollable, feared, involuntary exposure, inequitable distribution of risk, not easily reduced, catastrophic, risk increasing, fatal consequences, risk to future generations) and the 'Unknown' (not observable, risk unknown to science, delayed effect, new risk) [29]. People make judgements about the persuasiveness and trustworthiness of experts involved in communicating risk and find risks less acceptable if they believe that the communication of the risks between experts and the community is poor [30].

Research into decision-making under uncertainty showed that the rational subjective expected utility models which presumes that a good or 'rational' decision maker will sum the utilities and choose the action with the greatest total utility, does not explain how people make decisions [27,28]. Kahneman and Tversky's research confirmed findings from the subjective perception of risk and further contributed to an understanding of how information about risks or uncertainties of outcomes influences decisions [27,28]. Using hypothetical scenarios, studies have shown that people consistently underestimate risks of familiar and frequent events and overestimate the occurrence of low probability, but high consequence risks. Thus, rare events are perceived as more likely to occur than they do and common events are thought to occur less often than they do. The classic problems devised by Kahneman and Tversky involved asking subjects to choose between the certainty of saving 200 out of 600 lives or taking a 1 in 3 chance of saving 600 lives. They found that people's responses to possible negative outcomes are more extreme than their responses to possible positive outcomes. People also demonstrate a tendency to believe that their own risks are less than others, particularly if they believe that their exposure to risk is in some way under their control [31]. The impact of this tendency, described *as unrealistic optimism *and/or the *illusion of control*, is to reduce the perceived need to take protective measures [32].

Of particular importance to the decision to immunise are studies describing the operation of omission bias and choices involving ambiguous situations [33]. Omission bias describes a preference for taking no action if the action might cause harm, even if there is a greater risk of harm by 'doing nothing'. Ambiguity describes the decision-maker's feeling that there is missing information relevant to outcome or choice [33]. The effect of ambiguity on choice is to reduce people's willingness to act or to postpone the action until the missing information can be obtained [33]. These studies used hypothetical scenarios, making decisions about others, and participants were generally tertiary students and sometimes, parents.

As stated above the Health Belief Model by itself has been found wanting in terms of being able to explain parents' behaviour with respect to immunising their children. Conceptual pieces have been written describing how subjective risk perception and risky decision-making theories may be useful in understanding parents' behaviour and choices although these have not drawn on primary data [7]. Using a qualitative design, the aim of this study was to:

explore the salience of the decision-making and risk perception findings to parents' choices to immunise their young children;

examine the utility of the risk perception and decision-making theories to provide a more detailed explanation of the differences between immunisers' and non-immunisers' perceptions of severity, susceptibility to disease and benefits of vaccines; and

understand how these theories might explain perceptions of risk and reactions to a 'new strain of flu' for themselves and their children.

## Method

### Sampling

A stratified purposeful sampling strategy [34], was used to identify first time and experienced mothers of infants who were *completely immunised *(for age), *incompletely immunised *(behind the recommended immunisation schedule), *partially immunised *(parents chose or advised not to have a specific immunisation) or who had *no immunisations*. Initially, mothers of children between 14-16 months were approached. This age allowed a range of immunisation experiences to be discussed with participants while minimising the time since the first immunisation. To obtain sufficient numbers of non-immunisers and partial immunisers this age range was broadened to include 3 to 30 months. It was initially proposed that interviewing approximately 8 mothers from each immunisation category would be sufficient to discern patterns of similarity and difference between these categories. If preferred by the mother, both parents could participate in the interview.

### Participants

Possible participants were identified by Maternal and Child Health (M&CH) nurses in five metropolitan local government areas in Melbourne, Australia. Nurses were asked to approach mothers fitting the immunisation categories and to include, to the best of their knowledge, mothers of high and low education (< Year 11) and high and low income (held a Health Care Card). Parents were identified as fitting these categories from informal information available to the nurses. Parents from non-English speaking backgrounds whose English was poor were not interviewed. The Nursing Mothers Association group for the area also advertised the study.

Ethics approval was granted by the Royal Children's Hospital Ethics in Human Research Committee. Participation was voluntary, with written consent required. Participants provided informed consent to be interviewed, and for the interview to be taped. Participants were assured that they could stop the interview at any time, could choose not to answer any question if they didn't want to and that their responses would be confidential and transcripts anonymised. The interviewer did not have a dual relationship with the participants (i.e. she was neither a clinician nor provider of health services/care).

Recruiting and interviewing continued until 'saturation' occurred [34] (i.e. no new information was obtained from the interviews) for complete, incomplete and non-immunisers or until no more new parents fitting the categories could be identified, as was the case for partial immunisers.

Over the period of data collection, 94 families were identified as possible participants. Forty-eight interviews were arranged and 45 completed. One participant withdrew consent prior to the interview (she did not believe she had anything to say). Two participants were not at home at the time scheduled for the interview and neither returned follow-up phone calls. Of those not interviewed, 17 fitted categories for which a sufficient number of interviews had been conducted (complete immunisers); 11 were not interviewed due to language difficulties; 12 could not be contacted by the nurses and 6 refused. Interviews were undertaken in 1995-96. For six interviews both mother and father participated in the interviews (three of these were non-immunising families). All interviews were conducted in the participants' homes.

### Interview structure

Semi-structured, one-on-one interviews were used to collect information from parents about their children's health, the experience of illness in the family, their understanding and interpretation of risk and how all of these related to their decision to immunise. The method of one-on-one interviews rather than focus groups, was chosen as the aim was to explore the parents' experiences and path to choosing to immunise or not, rather than a group discussion of the pros and cons of immunisation. To understand the context of parents' decisions to immunise, the interviews covered four themes: (1) how mothers keep their children healthy; (2) experience, familiarity and concerns regarding both vaccine preventable diseases and other diseases; (3) concepts and influences on risk perception and (4) the decisions, experience and outcomes regarding immunisation. The interview began with questions about health as a non-threatening introduction and to place the consequent discussions about disease and disease prevention in the framework or context of health.

Questions about diseases and the family's experience of them were included to explore the relationship between common illnesses experienced by the family and vaccine preventable diseases. What was of interest here was which diseases were familiar, which were unfamiliar, which were to be avoided if possible and which were 'just' childhood illnesses.

### Interpretation of risk information and omission bias

To aid the investigation of how parents understand and interpret risk information the following two hypothetical news items about an influenza outbreak were read to the participants.

#### Radio news report 1

Health authorities issued a warning today about a new strain of flu expected this winter. The flu affects the airways, making breathing difficult and causing repeated bouts of coughing. Long term effects of pneumonia and brain inflammation have been reported in some cases. This strain appears to affect adults between the ages of 20-50 years. Several deaths occurred last year from the A-strain of this virus.

*Doctors have recommended that all adults should be vaccinated, especially those who are overworked, stressed and tired*.

#### Radio news report 2

Health authorities issued a warning today about a new strain of flu expected this winter. The flu affects the airways, making breathing difficult and causing repeated bouts of coughing. Long term effects of pneumonia and brain inflammation have been reported in some cases. Several deaths occurred last year from the A-strain of this virus. Many of those who died were children under 5 years. *Doctors have recommended all young children should be vaccinated*.

The description of symptoms and complications was taken from a description of the complications for pertussis [35]. The doctors' recommendations were written so that the parents could consider themselves 'at risk' in the 1st instance, parents of young children often feeling overworked and tired, and in the 2nd scenario their child/children fitted the 'at risk' group.

Omission bias was examined in this study by asking parents to respond to the following statement:

STATEMENT 1 Some people say they won't vaccinate because they would feel worse if their child died because of the injection than if the child was not immunised and died from the disease.

Participants were asked their opinion and were then read a second statement:

STATEMENT 2 Some people say they would vaccinate because they would feel worse if their child got the disease and died or was brain damaged when they could have had an injection to prevent it.

These statements were used rather than the more complicated scenarios developed by others (e.g. [33]) because it was believed they captured the essential element of omission bias in circumstances with which the parent could identify.

The interview concluded with discussions of the process of deciding to immunise or not and included a discussion of structural and non-structural barriers.

### Interview procedure

All interviews were conducted in the participants' homes at times convenient to them by the first author. Interviews lasted between 45 to 90 minutes. Socio-demographic information including family size and type (two or one parent family), mother's age, parental occupations and education levels was collected at the end of the interview. For those children who were immunised, immunisation status was determined from the immunisation records held by the parent. All interviews were audio-taped and fully transcribed. The interview focussed on the sole or youngest child in the family. Previous experience of disease and immunisations for older children was discussed in terms of its effect on decisions for the youngest child.

### Method of analysis

Interviews were thematically coded after all interviews had been collected. This analysis focussed on determining whether parents' descriptions of their experiences and beliefs were congruent or incongruent with theories of health behaviour, decision-making and risk perception. The coding was undertaken by the first author. No formal testing of the reliability of the coding was undertaken although discussions with colleagues about the analysis and the meanings and patterns derived from this were extensively undertaken.

## Results

Interviews were completed with 16 mothers whose children had completed immunisations appropriate for their age, 12 whose children were incompletely immunised, seven whose children were partially immunised (chose or advised not to have at least one component), and ten whose children had no immunisations. All families with incomplete immunisations had two or more children. (See Table 1.)

**Table 1:** Socio-demographic characteristics of the sample

	Immunisation status							
	
	Complete		Incomplete		Partial		None	
	
	*n *= 16	(%)	*n *= 12	(%)	*n *= 7	(%)	*n *= 10	(%)
Family size								

1st or only child	8	(50)	0	(0)	3	(43)	4	(40)

2 or more siblings	8	(50)	12	(100)	4	(67)	6	(60)

Income								

Health Care Card*	10	(63)	4	(33)	2	(29)	3	(30)

No Health Care Card	6	(38)	8	(67)	5	(61)	7	(70)

Maternal education level								

Did not complete secondary school	5	(31)	6	(50)	3	(43)	1	(10)

Completed secondary school	2	(13)	1	(8)	1	(14)	0	(0)

Post secondary school qualifications	9	(56)	5	(42)	3	(43)	9	(90)

*Indicator of low income

The following section presents a brief summary of similarities between immunisers and non-immunisers in terms of the concepts in the Health Belief Model. This is followed by a critical interpretation of the data linking this model with the theories of subjective perception of risk and decision-making under uncertainty. Finally the differences found between complete, incomplete, partial and non-immunisers in terms of these theories are summarised.

Table 2 summarises the differences and similarities between complete, incomplete and non-immunisers in terms of the core concepts of the Health Belief Model from these interviews (see [16] for further details). Partial immunisers formed two groups; those whose child had had a severe reaction to DTP (Diphtheria, Tetanus, Pertussis vaccine) (n = 3) where the parents had been advised not to continue with vaccination and those who chose to only undertake some vaccinations or changed their mind about vaccinations after the first DTP vaccine (n = 4). The former of these expressed views similar to complete immunisers and the latter to non-immunisers. To better understand these differences in perceptions, the interviews were analysed firstly for themes from the studies of risk perception-dread, familiarity and controllability.

**Table 2:** Summary of differences between complete, incomplete and non-immunisers* in terms of the Health Belief Model

	Severity	Susceptibility	Benefits	Barriers	Cues to action
Complete immuniser	Diseases serious, better to be prevented	Likely to get diseases	Vaccines are safe & effective	Lack of information about vaccines, diseases & side effects	Have health provider one can trust
					
			Serious side effects are rare		

Incomplete immuniser	Better to get either the vaccine or the disease when young (for some diseases)	Children susceptible to diseases/sickness in general ('they are always sick')	Vaccines are safe but not effective	Minor illnesses, forgetting, advice from health professionals	School immunisation certificate (but not for age-appropriate immunisations)
	
	Diseases are serious for adults	Adults more likely to have serious side effects from measles, rubella, mumps	Vaccines wont prevent diseases but will reduce effects	Confusion about which vaccines have been given	Health provider who understands family's circumstances

Non-immuniser	Diseases are not as serious as made out especially if child has healthy immune system	Susceptible to vaccine side effects	Vaccines cannot prevent diseases & are actively dangerous	Perceived serious consequences of vaccines	None

Common perceptions	Measles is common but rarely serious	Very young children are susceptible to vaccine side effects	None	Lack of information, trust, support	Trusted health provider and/or information
					
	Other diseases are serious but not common				

*3 Partial immunisers held views similar to complete immunisers and the other 4 held similar views to non-immunisers

### Dread of the unknown versus familiarity

Dread was an important determinant of what the participants in this study perceived as high risk. What was dreaded, however, differed between the immunisers and the non-immunisers. Immunisers dreaded the outcomes of the diseases, especially those with which they were unfamiliar. This fear motivated them to take the risk of immunising.

"The life threatening ones really concerned me, like ones I didn't know anything about...Polio really scared me and the thought of whooping cough... those are quite scary sort of concepts. Meningitis was frightening." (Complete immuniser, #11)

"I'm not sure about whooping cough, it just has horrible connotations in my mind but I'm not quite sure why, what can happen... Yeah, it's interesting isn't it, you know, people think it's the ones that you don't know about that you're likely to dismiss but it doesn't seem to me that way." (Complete immuniser, #13)

Polio, diphtheria, tetanus and meningitis were unfamiliar to these mothers but they conjured vivid images of severe outcomes. Of this group (immunisers), parents considered their children to be at greatest risk from meningitis. Even though most considered it unlikely that their children would contract these diseases, it was easy to imagine that if contracted, the worst was likely to happen.

LB: "If M hadn't been immunised, how likely do you think she would get these diseases?"

M: "Well I imagine it's fairly unlikely that she would get the diseases, um, but that's a double edged sword isn't it because having said that, that's largely because they've been for immunisation. ... I imagine it would be very worrying, particularly some of the worst ones, it would be quite frightening, and that's of course the reason that you give them [the immunisations.]" (Complete immuniser, #13)

The risks associated with vaccination were also perceived as being rare but rather than imagining the worst in this instance, they believed that one would be unlucky to have severe reactions.

Thus, on balance, the risk of not immunising was not worth taking and a 'common sense' approach was necessary. They likened vaccination to taking other safety precautions. In terms of the theories of risk, respondents were perceiving the diseases as less familiar therefore dreaded and unknown, and therefore possibly overestimating their risk, and perceiving vaccines as more familiar, and possibly underestimating their risk.

In contrast, non-immunisers dreaded the unknown or uncertain outcomes of the vaccines with major fears being for invisible/undetectable/distant problems such as the vaccines causing leukaemia, SIDS, AIDS and brain damage. For these parents, vaccines were not only ineffective but they were actively dangerous to children's health.

"Brain damage, affecting limbs. I've read there are long term effects which we really don't know about. There are new diseases coming up. Polio is no longer life threatening but there are cancers and AIDS, long term effects on the immune system. And this is because we are interfering, causing genetic changes." (Non-immuniser #26)

"They don't work and they do harm. Putting these things into their bodies-germs and all the other products-mercury aluminium etc cannot be good. It suppresses/disrupts the child's immune system. It doesn't work and it is harmful. It's not just the risk of the side effects but the long term effects that we don't know about now. Basically so many things which we did in the past we now know better and think were barbaric. I would rather not do something to my child when we don't know what the long term effects might be. There have been studies which have related these to leukaemia and other cancers, asthma eczema and all sorts of things. I don't want to do that to my child." (Non-immuniser, #19)

On the other hand, severe outcomes of the diseases were believed to be rare or only a problem for children with poor nutrition, poor sanitation, and compromised immune systems. Non-immunisers believed it was unlikely that their children would suffer serious complications if they contracted these diseases because they had healthy immune systems.

"If D did get one of these diseases it wouldn't necessarily be catastrophic. Some people do get very sick or die but what we don't know, what they don't tell us is that those children were probably not well to start with. Fairly sick children are more likely to get serious long term effects. Their health before the illness is crucial to how their bodies cope with the disease." (Non-immuniser,# 19)

"...a rejection of the notion that children have to be immunised against these diseases because the disease itself will automatically be worse than the immunisation and a concern that the you know the vaccination itself can have problems." (Non-immuniser,# 21)

For one non-immuniser, who was not 'against' using conventional medicine' she believed her children were protected from disastrous consequences because they had easy access to modern medical intervention.

"Well I just can't see the need. If your child catches measles and... that develops into anything else... we're not stuck in the middle of the country without good doctors or hospitals..." (Non-immuniser, #29)

As reported previously, both pro- and anti- immunisers were concerned about vaccines overloading even healthy but immature immune systems [16]

"I do sort of worry that we are vaccinating too much. I just worry about what it does to your immune system, to all our immune systems" (Complete immuniser, #14)

"He's still very thin but he's past the point of where I sort of see him [as] very vulnerable...like now I'm happy to give them to him." (Incomplete immuniser, #24)

"I mean I know she is a strong as a horse and I know she could have every shot under the sun and she'd be fine I just don't think it's a proper thing to do ... in only two-month olds." (Non-immuniser, #29)

### What is familiar is not dreaded

As would be expected from risk perception theory, diseases that were familiar to parents were not dreaded. Measles, mumps and rubella were not considered serious or life threatening by most parents irrespective of immunisation status. Most mothers were familiar with these diseases. They had had personal experience of these and remembered them as mild.

"[If] she happened to get measles, well I'm not that worried...because I had it and it was fine." (Complete immuniser, # 11)

The motivation to immunise against these diseases was, therefore, less than for diseases that were unfamiliar.

"...If it's a disease like measles, mumps, chicken pox, things like that you can let them get through fine, then if you got into meningitis, polio, well yeah, you'd have to think again." (Complete immuniser, #31)

"See, measles and German measles-I know that they brought the immunisation in because there are complications and there have been kids with complications. But see, like I remember from my generation a lot of us that was just the normal. Kids had the measles. So I am not so sure about those two whether it is important." (Incomplete immuniser, #15)

Measles, mumps and rubella were perceived as diseases that...'every child's got to get' and rather than avoid these diseases it was best to 'get them out of the way' as early as possible, especially as it was believed that these diseases were more serious in adults. There was no urgency in having their children vaccinated for these diseases.

### Controlling exposure or outcome

The idea of control was also used by parents to explain their choice to vaccinate. One explanation for immunising given by complete immunisers was that they could not control their children's exposure to diseases and hence, it was safer to vaccinate. By doing so they could control, to some extent, the diseases that their children were at risk of contracting.

"I think the world of her and I thought if [I] can prevent her getting any of these diseases I will. (Complete immuniser, #7)

Incomplete immunisers believed vaccination would contain or reduce the effects of disease rather than prevent it completely.

"...kids still get measles and mumps so that's the silly thing isn't it really? It's only to prevent it, it can't cure, do you know what I mean? I've heard of kids still getting measles." (Incomplete immuniser #30)

"But it doesn't prevent the flu, you still get the flu but not a strong dosage." (Incomplete immuniser, #44)

In contrast, non-immunisers talked about being able to control their children's environment and therefore their exposure to disease. This non-immunising mother spoke of her reasons for vaccinating the family dogs:

"...because I cannot control what they [the dogs] do and what they eat. I can control [child's name]." (Non-immuniser, #19)

### Decision-making under uncertainty

The explanatory power of ambiguity, outrage, omission bias and optimistic control was also examined in these interviews.

### Insufficient information-ambiguity or outrage

Perceived lack of information or insufficiency of information should either provoke outrage [30] or hesitation from acting [33]. Participants provided examples of both. Lack of information about susceptibility to vaccine side effects caused mothers in some instances to refrain from vaccinating their child or to hesitate about immunisation. One mother had hesitated to immunise her second child until she could be reassured that he would not have severe side effects from the vaccine. She had reason to believe he would be particularly susceptible to such side effects because he was 'not robust', he had many food allergies and his father had collapsed after immunisation as an infant. She expressed an equivalent concern about the child's susceptibility to disease especially as he had a school-aged sibling who could expose him to disease. The major reason for her hesitation was that no one had seriously considered her questions or considered her son's case on an individual basis.

"I want someone to look at him as an individual and I don't feel that they are the medical community....I don't want people making the decisions for me. ...I want that information available so that I can make an informed choice" (Non- immuniser, #18)

Being aware that children could react to the MMR vaccine but not being told what that reaction was or what to expect also caused some hesitation with some mothers. Some mothers hesitated about immunising against Hepatitis B which was at the time of the study recommended for 'at risk' groups. It was unclear to these mothers what this phrase meant and if it applied to their children.

"[I] believe he should have the hepatitis one 'cause if he comes in contact with another child that's got it, but they say he's not at risk... but what makes him not at risk to get it? So I've been umming and aahing whether to get that one."(Complete Immuniser, #10)

The reverse of hesitating to act because of insufficient information was shown by others who figuratively 'shut their eyes' to the information about vaccine risk because it was unsettling.

"I think honestly speaking, this sounds stupid, but I think well, I don't want to hear it [about side effects], because it scares me. I know it might be stupid because you think, well you know they're s'posed to have it but if you start thinking well, what if you know if this happens and that happens well, then you wont immunise your children, so, there's a risk I s'pose."(Incomplete immuniser, #42)

Another response to a perception of insufficient information was anger or outrage. During the interview some mothers apologised for not being better informed about diseases.

Others were angry at their lack of knowledge about diseases and vaccines. This anger was not directed at themselves but at unspecified others. Anger was more often expressed by non-immunisers who believed that drug companies and doctors knew vaccines were not safe but kept the information from the public.

### Omission bias

Whether parents choose not to act, when action may cause harm, was explored. This was done with the use of two statements-describing whether (1) it would be worse to have your child die due to your action (immunise) or (2) it would be worse due to inaction (die from disease) (see Methods section for statements). Both statements presented uncomfortable possibilities to parents.

"You can't ... I mean as far as I'm concerned you lose a child you lose it and it's painful either way I like to think that I've done the best I can to protect him from it um and if you know it's because of the injection well to some degree I'm fatalistic. I mean if it's meant to be it's meant to be. There's not much you can do about it but I would rather know that I've taken every precaution I can instead of you know leaving him open and susceptible to these things."

*LB: Some people say they would vaccinate because they would feel worse if their child died from an illness which they could have prevented*.

"Possibly I would sit in that category." (Complete immuniser, #1)

Most parents, irrespective of the immunisation status of their children identified more with the second statement, with only a few parents identifying with the first:

"I'd have to agree with that. I think if you've given birth to a perfect healthy child and then you've introduced foreign substances into their body which has then damaged them in some way, ah, yeah, I don't know, I don't think I could live with myself. Whereas if they've caught the disease that's kind of c'est la vie you know. I mean it's still awful. It's still a great tragedy, especially if you do lose them. But I think that's that. If you talk about metaphysics, I believe in metaphysics and all the rest of it, so I'd sort of say well, they're meant to be here, they're meant to experience it, they're meant to deal with it or not deal with it depending on what they're here for. So I have to take a philosophical approach. It'd be devastating." (Non-immuniser, #32)

Opposite to what would be predicted, many of the non-immunisers disagreed with the first statement and were adamant that this did not form part of their reason not to immunise.

FATHER: "I think you would be foolish to reach [that conclusion] I mean we're not foolish. I couldn't possibly say that I would be more comfortable with the you know..."

MOTHER: "The child dying or at least you know that he died from the disease."

FATHER: "Yeah a natural thing rather than induced. Yeah that's where the natural therapy philosophy goes too far...That would never be reason to [not immunise]."

MOTHER: "No, for not immunising him. Yes. I can't even relate to it as a distinction." (Non-immunisers #27)

The main reasons parents gave for not agreeing with the first statement was the perception that this scenario was unlikely to occur and, irrespective of immunisation status, most parents believed they had done everything they could to prevent disease. Thus, parents used their perceptions of the risks of the outcome of death from vaccine or the risk of getting the disease to explain their choice.

"No, well I'd feel, well I think that that's part of the risk, that there is a small risk that your child will have a reaction to the immunisation that's that's minimal compared to the risk of them getting the disease if you don't immunise so I'd always opt to immunise. (Complete immuniser, #5)

"I think you've got more, to me I think she's got more of a chance getting something not being vaccinated than, she's a healthy little girl isn't she?" (Complete immuniser,#7)

For those who agreed with Statement 1, they based this on their belief that there was a greater risk from vaccines so the first statement was the more likely scenario.

### Optimistic bias and the illusion of control

Participants' responses to the two hypothetical radio news items, was concordant with the theories that perception of risk may be influenced by an unrealistic optimism about one's own risks or unrealistic perception of control over one's life. The participants generally did not believe that they would be at risk from the flu. They believed themselves to be healthy, not susceptible to flu and that they were strong enough to fight it off:

"I tend to think that couldn't happen to me 'cause I'm young and healthy and couldn't possibly die of flu...Its a few cases [dying] and that happens." (Complete immuniser, #13)

Although the scenarios were written specifically so that those being interviewed fitted the 'at risk group', the participants did not identify with this group. They believed that the people who suffered serious consequences of flu were different to themselves. When they heard similar items on the news they assumed the people who were badly affected were old, frail, sick, had not been eating well, had poor immune systems, low resistance or were people who did not look after their health.

"It says doctors recommend all should be vaccinated especially those who are overworked, stressed and tired. Well of course they would be the ones whose immune systems would not cope." (Non-immuniser, #19)

All non-immunisers believed the risk of this hypothetical flu vaccine was higher than the risk of the disease but some immunisers also perceived high risk and little benefit from the vaccine. They related incidents of relatives who had bad experiences after receiving a flu injection. They believed that there were other ways of reducing the risk of flu such as taking supplements, improving lifestyle and avoiding people with the disease. Those who did believe their family to be at risk of this flu already received annual flu injections, with the occurrence of serious illness in these families prompting them to have flu vaccines.

Participants were also asked whether knowing someone who had the illness and had been very ill would affect their decision to immunise against this hypothetical flu. Again the response was that it would depend on the state of the friend's health and their habits: whether they were unhealthy or stressed, or careless of their health. While it would be more concerning to hear of a friend who was ill, most did not think it would mean they themselves were more at risk.

### Perceptions of risk: comparing children and adults

In the second news item the 'at risk' group was children under five. All parents stated this item of news would be of greater concern to them. Their first action however, would be to seek more information from their health advisers before immunising. The risk of their children becoming ill was more important than the risk to themselves and all believed that their responsibility as parents was to do everything they could to protect their children. This responsibility made it stressful to make decisions for children because 'you can't afford to make the wrong choice', and one can't take risks for one's children where one might take risks for oneself.

"...[I'm] not prepared to risk them, I can control my risks." (Non- immuniser, #29)

"You've got to take the risk to prevent them getting sick." (Incomplete immuniser, #20)

"Take all the risk factors out of it and make sure they have a good life." (Incomplete immuniser, #34)

"Because I am so much more protective of their health than mine. I am concerned that they don't have the option of making choices as easily as I do and that is why I would like to make informed choices. And I feel like if I make the wrong choice for yourself and that is something that I wear, I am responsible for it. If I make the wrong choice for them it is more serious." (Non-immuniser, #21)

There was however, a reluctance to immunise. Many believed there was an over-reliance on immunisations and antibiotics and that they would only immunise if the disease was widespread or local (Statewide). If it were not widespread it was not worth the risk of preventative medicine.

Table 3 summarises the factors found to influence the decision to immunise and corresponding aspects of the explanatory theories used as a theoretical framework for this study. In this table we aim to show how the information from risk perception and decision making under uncertainty allow for a greater explanation or point to more nuanced action. For example, consideration of unfamiliarity with diseases would be part of the Health Belief Models framework of considering perceived severity. However the Health Belief Model does not explain or allow us to understand what people perceive as familiar or unfamiliar. One might think that if parents are not familiar with a disease they may not think it is serious, whereas subjective perception of risk would indicate that the reverse may be operating: unfamiliarity increases people's perception of risk. Similarly, where parents don't think their child is susceptible to the disease (a Health Belief Model construct), the idea of having 'optimistic control' helps to explain why they might think this.

**Table 3:** Factors influencing the decision to immunise drawing on the Health Belief Model, subjective perception risk and risky decision-making theories

	Explanatory theories	Component of theory
Factors prompting immunisation		

Unfamiliarity of disease	Health Belief Model	perceived severity
	
	Subjective perception of risk	dread/unfamiliarity

Safe and effective vaccines	Health Belief model	perceived benefits

Healthy, robust, resilient child	counter to Health Belief Model	perceived susceptibility

Not control/contain disease	Health Belief model	perceived severity
	
	Subjective perception of risk	uncontrollable outcome

Trust in institution offering vaccination	Subjective perception of risk	trust mediates perception, outrage

Factors causing hesitation		

Question need to prevent familiar disease	Subjective perception of risk	familiarity

Question child's resilience to side effects	Health belief model	perceived barriers

Negative impact on immune system	Health belief model	perceived barriers

Question vaccine efficacy	Health belief model	perceived benefits

Those at risk of serious illness are	Health Belief Model	perceived susceptibility

different to my child	Decision making	optimistic control

Structural barriers to completion		

Continual minor illness	Health belief model	perceived barriers

Poor information	Health belief model	perceived barriers
	
	Decision making	ambiguity

Poor communication	Health belief model	perceived benefits
	
	Subjective perception of risk	outrage
	
	Decision making	ambiguity

Knowing schedule and organisation	Health belief model	perceived barriers

Occurrence of serious side effects	Health belief model	perceived barriers

Factors prompting non-immunisation		

Dread of the unknown vaccine side effects	Subjective perception of risk	dread of unfamiliar
		
		uncontrollable outcome

Not safe nor effective	Health belief model	perceived benefits

Negative impact on immune system	Health belief model	perceived barriers

Safer methods to control disease	Decision making	optimistic control

### The meaning of numbers-parents as lay epidemiologists

In considering the news reports respondents were asked how many would 'several deaths' be to cause them to worry about the risks of the disease. It was difficult for the participants to respond to this question meaningfully and the production of these numbers was somewhat arbitrary; most were not comfortable giving their response, and many could not say. For instance, one couple said they could give an answer if it was needed to meet the research requirements but it would be meaningless. The numbers given varied from only one or two deaths, five in the State, or between one and ten percent. Others gave figures of more than fifty percent of those who got the disease would have to die for them to be concerned.

The question was useful, however, because it provoked participants to define the type of information they wanted in order to make sense of reports such as those they had just heard. For instance, they said their response to the number of deaths would depend on how similar to their own circumstances were those who had died. Did they live in Australia, Victoria or developed countries? Were those who were dying, previously healthy people or those who were sick and thus, more susceptible? Was it a familiar disease (flu) or rare (Ebola virus)? If it was unfamiliar it was frightening. If it was familiar (flu) they wanted to know the details of who it was who had died or suffered complications. Thus, it was not the statistics that were important for deciding on risk, but the characteristics of those who had the disease and the familiarity or unfamiliarity of the disease.

## Discussion

The decision to immunise or not is complex with perceptions of risks of vaccines, diseases and robustness of the child's health to be considered. In this study we have identified and clarified differences in perception between complete, incomplete and non-immunisers and also identified similarities between all mothers with respect to the decision to immunise their young children.

While the decision to immunise young children can be understood to some extent in the context of perceptions of severity and susceptibility to disease and benefits and barriers to immunisations, the theories of risk perception and decision-making add a depth of understanding to the differences found between these parents in terms of their perceptions and interpretations of what is risky and what is not. Two aspects in particular appear to be important: the familiarity or unfamiliarity with the disease and perceived control over risks or outcomes. Thus, perceptions of severity of disease are influenced by the unfamiliarity of the disease and/or the perception that these diseases will have uncontrollable outcomes. That is, diseases with which parents are least familiar are perceived as more severe than those with which parents are more familiar and therefore worth taking preventive action. Being a familiar disease contributed to delays in immunisation, or not immunising as these familiar diseases were not considered to be severe.

This finding is congruent with other primary studies. For example, Hilton et al [36] reported 'of all the diseases... measles was the one that parents most commonly reported having as a child. ...Indeed their experience of measles often rendered it a *less *threatening disease.... While parents with no experience of measles entertained the long-term damage it could inflict, those with experiences of it tended to minimise the risks' (p 174, authors' italics). From this it would appear that it is too simplistic to attribute reduction in immunisation uptake to a growing lack of familiarity with diseases because of the success of the immunisation programmes.

Understanding people's perceptions of what can and cannot be controlled is important to understanding their behaviour. There is both an aspect of fearing uncontrollable or unknowable outcomes and therefore taking preventive action and an optimistic belief or an illusion that the environment or risks can be controlled. Unlike the non-immunisers in this study and others [21], immunisers choose immunisation because they believe they have limited control over their children's environment and contacts. Many believed they could control risks to their own health or were willing to 'take the risk' with their own health.

Parents were less willing to take risks with their children's health than with their own. This was partly because children were perceived as more susceptible than healthy adults and partly because their children were dependent on them making good decisions. This unwillingness to take risks included being cautious of preventive action as well as cautious about diseases. An important barrier to action was the tension between what is 'natural' and medical intervention. For many mothers there was something 'unnatural' about medical intervention. They held a belief that medical intervention was necessary for important diseases but that it was not safe or necessary to use for all problems. Again, this perception that what is unnatural is more risky is congruent with the studies of subjective risk perception, but could not be predicted from the Health Belief Model.

Importantly, the participants believed that the risks of diseases and complications from disease were not equally spread throughout the community. When listening to reports of epidemics, it is not the number of people who are affected but the familiarity or unfamiliarity of the disease and the characteristics of those who had the disease that caused parents to worry about taking preventive action. Poor information or communication creates barriers to immunisation completion which can be understood in terms of the concepts of outrage and ambiguity. Lack of trust and poor communication between providers and parents exacerbated the belief that information was being kept from them. Because they all believed that parents were ultimately responsible for their children, this feeling that information was denied them frustrated and angered them.

### Limitations

This study has used qualitative methods to determine if aspects of theories of risk and decision-making can help to explain parents' decisions about immunising their children. Traditionally qualitative research has been associated with the generation, rather than the testing, of hypotheses, however, this denies a major strength of qualitative research which is to examine theories in the light of data. As such it is a method suited to producing understanding and to generating solutions to problems [34]. We believe this method, therefore, is appropriate to provide a greater understanding of complexities of decision making and perceptions of disease and vaccines and a depth of information not available from large scale quantitatively based surveys. The benefits of using the qualitative method described in this study is the large quantity of detailed information it provides, however, using a small non-randomly selected sample can present problems in determining the generalisabilty of the ensuing information. The results of this study confirm, complement and extend the findings of other studies in this area [4,5,36,37].

The data that this paper draws on were collected in the late 1990s and some may wonder at their currency. We believe that this might be a problem if the focus of the paper was about which diseases or vaccines are an issue; with scares and controversies these can change over time. The point of this paper however, has been to examine the utility of synthesizing theories of health protective behaviours, risk perception and decision-making. While we recognise that different socio-temporal contexts may create different issues (e.g. the impact of the MMR controversy was substantially greater in the UK than Australia), we argue that the approach we have taken in this paper provides a framework for us to make sense of people's reactions to and perceptions of old (and new) diseases and vaccinations at any time. We therefore think this work can contribute to, and be of particular importance in informing the public health approaches to new flu epidemics. It provides data supporting commentary and critique of the current public health approach to the issues of vaccine risk and immunisation uptake, being that continued provision of better risk information is not the answer [7].

We have found and would argue that the theories of risk perception and aspects of decision-making under uncertainty have been useful for understanding the differences and similarities between pro-immunisers and non-immunisers, except for the issue of omission bias. Parents in this study, whether immunisers or not, generally did not agree that a negative outcome was preferable or more acceptable from inaction. This finding raises some doubts about the methods and/or generalisability of findings from studies of this phenomenon, which usually involve multiple, similar, hypothetical situations with limited contextual information, presented in mathematical and probabilistic language. Participants in this study responded to these omission bias statements by generally denying that either contributed to their decision to immunise their child.

### Implications for communicating information about vaccines and diseases

Similar findings to this study have been reported by others [3-5,37] and have important implications for how public health addresses the issues of trust and communicates risk information. As others have noted there is more to risk communication than providing more facts about risks [3,4,7,23]. To paraphrase Hobson-West, from these studies and critiques, it is clear that education is *not *the main policy tool and ignorance is *not *the main enemy for maintaining immunisation uptake (p 279 [23]).

Drawing on the aspects of subjective perceptions of risk and decision-making under uncertainty, we believe the following needs to be considered in communicating risk information and health messages:

Facts and figures are not interpreted or acted upon rationally: dread, catastrophic potential and familiarity with the risk influences interpretation and action

People act as lay epidemiologists. Thus providing information about risks as though everyone has the same risk makes the advice unbelievable and can be discounted

Parents are more willing to take risks about their own health than with their children's health but this greater caution about their children's health does not automatically mean they will accept medical intervention

From the theory of subjective perception of risk people may well be wary of novel vaccines or therapies (manmade versus natural risks) hence there may be some hesitation in the uptake of such vaccines

People will discount their risks-'the people affected are not like me'. This may have implications for how people will assess their risk of being badly affected by any outbreak of new strains of influenza such as H1N1.

Clear communication which involves listening to, and not dismissing people's concerns, is valued.

## Conclusion

This study has shown that there is a need to understand and take into account how people subjectively perceive risks and how that influences their decision-making, in order to understand their choices and behaviours. Theories of risk perception and decision-making can help to explain differences in perceptions of severity and susceptibility of diseases and vaccines. Importantly, this study has found that health messages about the risks of disease which are communicated as though there is equality of risk in the population may be unproductive as these messages are perceived as unbelievable or irrelevant. The findings from this study have implications beyond the issue of childhood vaccinations as we grapple with communicating risks of new epidemics. Using and developing a more complex theoretical approach to public health issues may increase the likelihood we can understand the barriers to action and develop effective methods of communicating risk and delivering acceptable public health interventions. And indeed may usefully contribute to the current debates, especially in the UK, of how these theories of risk and decision-making can be used to 'nudge' other health behaviours [38,39].

## Competing interests

LB holds CSL Ltd shares. TN is chair of the Australian Technical Advisory Group on Immunisation and undertakes clinical trials of new vaccines.

## Authors' contributions

LB and TN contributed to the design of the study. LB conducted the interviews and undertook the analysis. Both contributed to drafting the paper and read and approved the final manuscript.

## Pre-publication history

The pre-publication history for this paper can be accessed here:

http://www.biomedcentral.com/1471-2458/11/943/prepub
